# Moderate increase of serum uric acid within a normal range is associated with improved cognitive function in a non-normotensive population: A nationally representative cohort study

**DOI:** 10.3389/fnagi.2022.944341

**Published:** 2022-09-01

**Authors:** Jinqi Wang, Rui Jin, Zhiyuan Wu, Yueruijing Liu, Xiaohan Jin, Ze Han, Yue Liu, Zongkai Xu, Xiuhua Guo, Lixin Tao

**Affiliations:** ^1^Beijing Municipal Key Laboratory of Clinical Epidemiology, Department of Epidemiology and Health Statistics, School of Public Health, Capital Medical University, Beijing, China; ^2^Department of Public Health, School of Medical and Health Sciences, Edith Cowan University, Perth, WA, Australia

**Keywords:** cognitive trajectory, cognitive function, serum uric acid, changes in serum uric acid, hypertension, cohort study

## Abstract

**Background:**

Associations between serum uric acid (SUA) and changes in cognitive function are understudied in non-normotensive populations, and many previous studies only considered the baseline SUA at a single time point. We aimed to examine the effects of baseline SUA and 4-year changes in SUA on cognitive changes in the non-normotensive population.

**Materials and methods:**

In the China Health and Retirement Longitudinal Study (CHARLS), cognitive function was measured based on executive function and episodic memory in four visits (years: 2011, 2013, 2015, and 2018). We identified two study cohorts from CHARLS. The first cohort included 3,905 non-normotensive participants. Group-based single-trajectory and multi-trajectory models were applied to identify 7-year cognitive trajectories. Adjusted ordinal logistics models were performed to assess the association between baseline SUA and 7-year cognitive trajectories, and subgroup analyses were conducted according to the presence of hyperuricemia or SUA levels. The second cohort included 2,077 eligible participants. Multiple linear regression was used to explore the effect of a 4-year change in SUA on cognitive change during the subsequent 3-year follow-up.

**Results:**

Four distinct single-trajectories of global cognitive performance and four multi-trajectories of executive function and episodic memory were identified. Higher baseline SUA levels were significantly associated with more favorable cognitive single-trajectories (OR_*Q*4 vs_. _Q1_: 0.755; 95% CI: 0.643, 0.900) and multi-trajectories (OR_*Q*4 vs_. _Q1_: 0.784; 95% CI: 0.659, 0.933). Subgroup analyses revealed that the protective effect of SUA was significant in the non-hyperuricemia groups or the low-level SUA groups. Additionally, changes in SUA could influence future cognitive changes. Compared with non-hyperuricemia participants with elevated SUA, non-hyperuricemia participants with decreased SUA and patients with persistent hyperuricemia had a higher risk for cognitive decline. Furthermore, only the Q3 group of changes in SUA could enhance global cognitive function compared with the Q1 group (β: 0.449; 95% CI: 0.073, 0.826).

**Conclusion:**

Our study indicates that the maintenance of normal SUA levels and a moderate increase of SUA were advantageous in improving cognitive function or trajectories in a non-normotensive population. Conversely, SUA may impair cognitive function in patients with persistent hyperuricemia.

## Introduction

Cognitive impairment and dementia have become major and increasing global health challenges that reduce the quality of life and cause a huge economic burden (Jia et al., 2018; Nichols et al., 2019; Davis et al., 2022). Due to the aging population, the global number of people living with dementia or cognitive impairment has dramatically increased (Nichols et al., 2019). Approximately, more than 15% of Chinese older people are suffering from cognitive impairment (Xue et al., 2018). Several studies suggested that people with hypertension, even under prehypertensive conditions, were more likely to have cognitive decline or dementia (Gottesman et al., 2014; Chen et al., 2015; Iadecola and Gottesman, 2019; de Menezes et al., 2021). Considering that there is no effective disease-modifying treatment for cognitive impairment or dementia (Li et al., 2020; Davis et al., 2022), early identification of possible modifiable risk factors of rapid cognitive decline is crucial to prevent or delay the incidence of cognitive impairment among the prehypertensive and hypertensive populations.

As a natural antioxidant, serum uric acid (SUA) is thought to exert a neuroprotective effect by acting as a free radical scavenger to prevent the development of cognitive dysfunction (Bowman et al., 2010; Tuven et al., 2017; Kim et al., 2020). However, urate-lowering therapy plays an important role in the management of hypertension to prevent diabetes, chronic kidney disease, stroke, and adverse cardiovascular outcomes (Kawai et al., 2012; Sharaf El Din et al., 2017; Cassano et al., 2020; Virdis et al., 2020). Hypertension with other chronic diseases co-occurring in the same individuals may result in a more severe and accelerated decline in cognitive function (Iadecola and Gottesman, 2019). Previous epidemiological studies and meta-analyses found the association of the lower SUA level with poorer cognitive function and dementia (Wang F. et al., 2017; Wang T. et al., 2017; Huang et al., 2019; Scheepers et al., 2019; Chen et al., 2021; Zhou et al., 2021; Huang Y. et al., 2022), but whether SUA plays a neuroprotective role among prehypertensive and hypertensive populations remains unclear. Additionally, the potential protective effects of SUA on cognition were controversial in other studies. The opposite association (Beydoun et al., 2016; Suzuki et al., 2016; Latourte et al., 2018b; Singh and Cleveland, 2018; Alam et al., 2020) or no association (Richard et al., 2021) between SUA and cognitive performance have been reported. As for the reason, most previous studies included participants with hyperuricemia. Patients with hyperuricemia may have different underlying etiology pathways due to the impact of higher levels of SUA on adverse health outcomes as compared to the non-hyperuricemia population (Huang Y. et al., 2022). The different effects of SUA on cognition among both non-hyperuricemia and hyperuricemia populations still need further exploration.

Several other limitations were present in previous studies. Most previous studies only recorded the SUA levels at baseline, failing to take into account the potential effect of changes in SUA in follow-up on subsequent cognitive function (Qiao et al., 2021). Moreover, these studies neglected the longitudinal change pattern of cognitive function during the follow-up period. An advanced statistical method, group-based trajectory models (GBTM), allowed us to identify subgroups that share common developmental trajectories over time (Nagin and Odgers, 2010).

Thus, the present study was conducted in the non-normotensive population, including prehypertensive and hypertensive individuals, to: (1) identify different 7-year cognitive trajectories and investigate the association between the baseline SUA levels and cognitive trajectories; (2) assess the above associations among both the non-hyperuricemia and hyperuricemia populations; and (3) examine the effect of 4-year changes in SUA on subsequent 3-year changes in cognitive performance.

## Materials and methods

### Study population

China Health and Retirement Longitudinal Study (CHARLS) is a nationwide survey that was conducted among Chinese adults aged 45 years or older (Zhao et al., 2014), which is a prospective cohort collecting data on social and economic information, anthropometric and laboratory measurements, demographic characteristics, health-related behaviors, and health conditions. A total of 17,707 participants from 150 county-level units distributed in 28 provinces of China were recruited at baseline (wave 1: 2011–2012), and the subsequent three follow-up visits were carried out (wave 2 in 2013, wave 3 in 2015, wave 4 in 2018). Details about the data were previously described in an earlier publication (Zhao et al., 2014). The protocols of CHARLS were approved by the Biomedical Ethics Review Committee of Peking University (IRB00001052-11015). All participants provided their signed informed consents. We identified two study cohorts from CHARLS.

The first cohort was constructed to explore the longitudinal association between baseline SUA and 7-year cognitive trajectories. Participants were excluded if they had missing data on gender, age, SUA, and cognitive function; had self-reported the previous diagnosis of brain damage, intellectual disability, and memory-related disease (Alzheimer’s disease, brain atrophy, Parkinson’s disease); or were normotensive at baseline in 2011. Finally, a total of 3,905 participants with two or more subsequent follow-up visits were enrolled for the analyses (Supplementary Figure 1).

The second cohort was constructed to examine the effect of changes in SUA on the future 3-year cognitive changes. We calculated changes in SUA using the SUA levels in 2011 and 2015 to predict the risk of developing cognitive decline from 2015 to 2018. Participants were excluded if they had missing data on gender, age, SUA, and cognitive function at baseline or the survey in 2015; had self-reported the previous diagnosis of brain damage, intellectual disability, or memory-related disease; or were normotensive in or before 2015. Ultimately, a total of 2,077 participants who attended the last survey in 2018 (wave 4) were enrolled in the study (Supplementary Figure 2). The timeline of the study is presented in Supplementary Figure 3.

### Cognitive function

Cognitive function was assessed by two measures: episodic memory and executive function according to previous studies (Lei et al., 2012; Li et al., 2020; Huang Y. et al., 2022). In the episodic memory test, participants were instructed to recall words immediately (immediate recall) and 5 min later (delayed recall) after examiners read 10 Chinese words to them. The number of correctly recalled words was scored. The episodic memory score was the average score of the immediate and delayed recall tests and ranged from 0 to 10 (Hua et al., 2020). In the executive function test, participants were shown a figure and asked to redraw it. If the participant succeeded, the score was 1. They also were asked to subtract 7 from 100 serially five times and to identify the date (month, day, and year), season, and day of the week. Answers to these questions were summed into the executive function score ranging from 0 to 11 (Wu et al., 2021a). The global cognition score was calculated as the sum of episodic memory score and executive function score and ranged from 0 to 21.

### Measurements of serum uric acid and serum uric acid changes

Venous blood was collected from participants with their fasting status recorded. The blood samples were first kept in local hospitals, then transported to Peking University in Beijing, and stored at -80°C until measurement. The level of SUA was analyzed using the UA Plus method (Wang T. et al., 2017; Chen et al., 2019). Hyperuricemia was defined as SUA > 6.0 mg/dL in women and > 7.0 mg/dL in men (Huang Y. et al., 2022). Sex-specific quartiles of baseline SUA were constructed and used for subsequent analysis.

Participants were divided into five categories according to the changes in SUA from baseline in 2011 to the follow-up survey in 2015: “non-hyperuricemia with elevated SUA,” “non-hyperuricemia with decreased SUA,” “incident hyperuricemia,” “remittent hyperuricemia,” and “persistent hyperuricemia.” “Non-hyperuricemia with elevated SUA” was defined as hyperuricemia absent both in 2011 and 2015 and an increase in SUA levels. “Non-hyperuricemia with decreased SUA” was defined as hyperuricemia absent both in 2011 and 2015 and a decrease in the SUA levels. Remittent hyperuricemia was defined as hyperuricemia that was present only in 2011 but not hyperuricemia in 2015. Incident hyperuricemia was defined as hyperuricemia that was present only in the follow-up survey in 2015. Persistent hyperuricemia was defined as hyperuricemia present both in 2011 and 2015 (Liu et al., 2018). The first group was set as the reference group. Then, changes in SUA were calculated as the SUA level in 2015 minus that in 2011: *ChangesinSUA* = *SUA*_2015_−*SUA*_2011_. Both continuous and categorical (quartiles) types were constructed and used in analyses.

### Measurements of non-normotensive status

Systolic blood pressure (SBP) and diastolic blood pressure (DBP) were measured three times repeatedly by trained medical staff using an Omron HEM-7200 Monitor. We used the average of the three measured results as the final blood pressure. Past medical history and medication history of hypertension were obtained from self-reports.

The non-normotensive status included prehypertensive and hypertensive statuses (Wu et al., 2021b). Hypertension status was defined as SBP ≥ 140 mmHg or DBP ≥ 90 mmHg, the use of anti-hypertensive medication, or self-reported history of hypertension (Chobanian et al., 2003). Prehypertension status was defined as SBP of 120–139 mmHg or DBP of 80–89 mmHg (Chobanian et al., 2003).

### Covariates

Several covariates were included in our analyses. Sociodemographic characteristics included age, gender (men and women), marital status (married and others), and educational level (no formal education, junior high school or below, and high school or above). Health-related lifestyles included self-reported smoking status (non-smoker, former smoker, and current smoker) and drinking status (drink more than once a month, drink but less than once a month, and none of these). Body mass index (BMI) was calculated as weight in kilograms divided by height in meters squared and was categorized as follows: < 18.5, 18.5–23.9, ≥24.0 (Wang and Zhai, 2013). Depressive symptoms were assessed using the 10-item Center for Epidemiological Studies Depression Scale (CES-D-10). A score of ≥ 12 indicated the presence of depressive symptoms (Chen and Mui, 2014). Self-reported history of cardiometabolic diseases (CMD) included diabetes, dyslipidemia, stroke, and heart-related diseases (Huang Z. T. et al., 2022). We calculated the estimated glomerular filtration rate (eGFR) using the Chronic Kidney Disease Epidemiology Collaboration’s 2009 creatinine equation (Levey et al., 2009). Finally, physical activity was assessed by the questionnaires among a randomly selected subsample of the study population. Physical activity was defined as ≥150 min/week of moderate, or ≥ 75 min/week of vigorous activity, or a combination (≥600 metabolic equivalents [METs]) (Lloyd-Jones et al., 2010; Ding et al., 2020). Otherwise, they were categorized as physically inactive.

### Statistical analysis

Baseline characteristics were presented as the mean (standard deviation, SD), median [interquartile range, IQR], or number (percentage), as appropriate.

Single trajectories of global cognitive function were determined using GBTM to map the developmental course of cognitive performance from the baseline survey to three follow-up visits. The jointly longitudinal changes in episodic memory and executive function over time were estimated using group-based multi-trajectory modeling (GBMTM). GBMTM is a new application of GBTM and allows the joint modeling of the trajectories of multiple outcomes (Nagin et al., 2018). GBTM and GBMTM can identify clusters of individuals following similar change patterns through multiple visits. Varied models were considered to choose the optimal number of distinct groups and trajectory shape parameters (e.g., linear, quadratic, and cubic) based on Bayesian information criteria (BIC), Akaike information criterion (AIC), and average posterior probabilities (APP) of each trajectory group (≥0.70) (Li et al., 2020). In addition, the sufficient sample size in each trajectory group (>5% of the sample) and clinical interpretation are important elements when determining the best model. Finally, trajectories of cognitive function were plotted over a 7-year follow-up time.

Ordinal logistics regression models were used to assess the associations between baseline SUA and 7-year cognitive trajectories. The final multivariable model was adjusted for age, gender, marital status, education level, smoking status, drinking status, BMI, depressive symptoms, CMD, and the presence of prehypertension at baseline. We evaluated the dose–response relationship between SUA, as a continuous change, and each trajectory of cognitive function using restricted cubic splines (RCS) with four knots (at the 5th, 35th, 65th, and 95th percentiles). Then, subgroup analyses based on the hyperuricemia status were performed. Considering the relatively small sample size of the population with hyperuricemia (*N* = 262), other subgroup analyses by the SUA levels were conducted to test the robustness of the results. Participants were divided into the high-level SUA and low-level SUA groups based on the sex-specific median and 75th quartile of SUA. Both continuous and categorical (sex-specific median of SUA in each subgroup) types were constructed and used in subgroup analyses (lower SUA level as a reference category).

Subsequently, we used multiple linear regression models to explore the effect of changes in SUA on 3-year cognitive change, adjusting for the same abovementioned covariates and SUA in 2015. The regression coefficient (β) and its 95% confidence interval (CI) were presented. The cognitive change was calculated by differences in cognitive scores between 2015 and 2018:*Cognitivechanges* = *cognitivescore*_2018_−*cognitivescore*_2015_. RCS with four knots was used to capture the dose–response relationship between changes in SUA and cognitive changes.

Sensitivity analyses were also conducted as follows: (1) eGFR was further adjusted to explore the stability of our findings; (2) the models were further adjusted for physical activity in the subpopulations of two study cohorts who underwent physical activity assessments; (3) the generalized estimating equation (GEE) models were used to examine the longitudinal association between the SUA levels and cognitive function in the following several years; (4) the effect of baseline SUA on cognitive trajectories in subgroups, according to age, gender, depression status, and the presence of prehypertension and non-normotensive combined with other CMD were analyzed; (5) cognitive scores in 2018 were used as the secondary outcome to explore the effect of changes in SUA on cognitive function, adjusting for the abovementioned covariates plus cognitive scores in 2015.

Group-based trajectory models and GBMTM techniques were implemented using the Proc Traj in Stata software version 15.1. Other statistical analyses above were performed with the R software version 4.1.0. A two-sided *p*-value < 0.05 was considered statistically significant.

## Results

### Baseline characteristics

The mean age of the 3,905 participants was 58.48 ± 8.54 years and 50.4% of participants were men. The mean age of the 2,077 participants was 57.78 ± 8.06 years and 50.4% of participants were men. The distribution of baseline SUA, changes in SUA, baseline covariates, and cognitive scores is shown in Table 1.

**TABLE 1:** Baseline characteristics of the study population in two cohorts.

Characteristics	The first cohort	The second cohort
No. of participants	3905	2077
Age(years), mean (*SD*)	58.48 (8.54)	57.78 (8.06)
Male, *n* (%)	1968 (50.4)	1046 (50.4)
Married, *n* (%)	3383 (86.6)	1833 (88.3)
**Educational level, *n* (%)**		
No formal education	1400 (35.9)	654 (31.5)
Junior high school or below	1997 (51.1)	1136 (54.7)
High school or above	508 (13.0)	287 (13.8)
**Smoking status, *n* (%)**		
Non-smoker	2309 (59.1)	1243 (59.8)
Former smoker	391 (10.0)	206 (9.9)
Current smoker	1205 (30.9)	628 (30.2)
**Drinking status, *n* (%)**		
More than once a month	1072 (27.5)	569 (27.4)
Less than once a month	312 (8.0)	177 (8.5)
None of these	2521 (64.6)	1331 (64.1)
Depressive symptoms, *n* (%)	875 (22.4)	504 (24.3)
**BMI (kg/m^2^), *n* (%)**		
<18.5	160 (4.1)	81 (3.9)
18.5–23.9	1829 (46.8)	897 (43.2)
≥24.0	1916 (49.1)	1099 (52.9)
**CMD, *n* (%)**		
Diabetes	295 (7.6)	154 (7.4)
Dyslipidemia	510 (13.1)	273 (13.1)
Stroke	76 (1.9)	31 (1.5)
Heart-related diseases	559 (14.3)	295 (14.2)
At least 1 CMD, *n* (%)	1080 (27.7)	573 (27.6)
Prehypertension, *n* (%)	1596 (40.9)	1011 (48.7)
eGFR (mL/min/1.73 m^2^), mean (*SD*)	91.97 (14.86)	92.77 (14.43)
SUA (mg/dL), mean (*SD*)	4.61 (1.29)	5.11 (1.41)
**Cognitive scores, mean (*SD*)**		
Global cognitive function	12.23 (3.50)	12.32 (3.57)
Executive function	8.45 (2.55)	8.54 (2.56)
Episodic memory	3.78 (1.67)	3.79 (1.67)
**Changes in SUA**	–	
No hyperuricemia and increased SUA	–	1258 (60.6)
No hyperuricemia and decreased SUA	–	499 (24.0)
Incident hyperuricemia	–	199 (9.6)
Remittent hyperuricemia	–	50 (2.4)
Persistent hyperuricemia	–	71 (3.4)
Changes in SUA (mg/dL), mean (SD)	–	0.57 (1.10)

Data are presented as the mean (SD), median [IQR] or number (%), as appropriate. BMI, body mass index; CMD, cardiometabolic diseases; eGFR, estimated glomerular filtration rate; SUA, serum uric acid.

### Estimated cognitive trajectories

**FIGURE 1:**
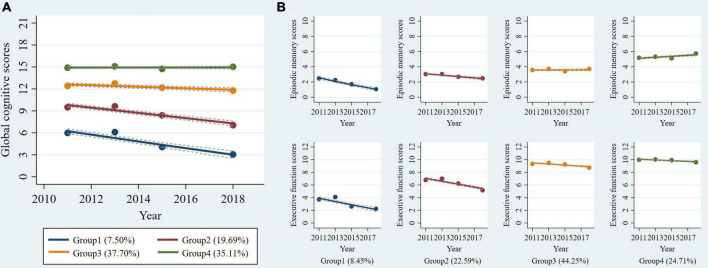
Trajectories of cognitive function from 2011 to 2018 among non-normotensive populations. Graphs show four trajectories of global cognitive function **(A)** and four multi-trajectories of executive function and episodic memory **(B)**. Descriptions of the trajectory groups are as follow: **(A)** group 1: “low-declining,” group 2: “moderate low-declining,” group 3: “moderate high-stable,” group 4: “high-stable”; **(B)** group 1: “episodic memory: low-rapid declining + executive function: low-declining,” group 2: “episodic memory: low- minimal declining + executive function: moderate-declining,” group 3: “episodic memory: moderate-stable + executive function: high-declining,” group 4: “episodic memory: high–rising + executive function: high-stable.”

Supplementary Table 1 shows the procedure of choosing the optimal group number and shape parameter for the final single-trajectory model. The BIC was lower for the model with six trajectories (BIC = -33582.86). However, APP was less than 0.7 for the two trajectories group. Additionally, the average APP of all groups for the model with five trajectories was less than with four trajectories. Thus, we determined four single trajectories of global cognitive function reflected the longitudinal change patterns over 7 years (Figure 1A): class 1, “low-declining” (*n* = 293, 7.50%); class 2, “moderate low-declining” (*n* = 769, 19.69%); class 3, “moderate high-stable” (*n* = 1,472, 37.70%); and class 4, “high-stable” (*n* = 1,371, 35.11%). The four groups represented a trend of increasingly better cognitive trajectories.

Using the same criteria and procedure (Supplementary Table 2), we determined four groups using GBMTM that showed the longitudinal joint changes of episodic memory and executive function (Figure 1B): class 1, “episodic memory: low-rapid declining + executive function: low-declining” (*n* = 330, 8.45%); class 2, “episodic memory: low-minimal declining + executive function: moderate-declining” (*n* = 882, 22.59%); class 3, “episodic memory: moderate-stable + executive function: high-declining” (*n* = 1,728, 44.25%); class 4, “episodic memory: high-rising + executive function: high-stable” (*n* = 965, 24.71%). The four groups also can represent a trend of increasingly better cognitive multi-trajectories. The maximum likelihood estimates for the final trajectory models are summarized in Supplementary Tables 3, 4.

### Associations between the baseline serum uric acid and 7-year cognitive trajectories

**FIGURE 2:**
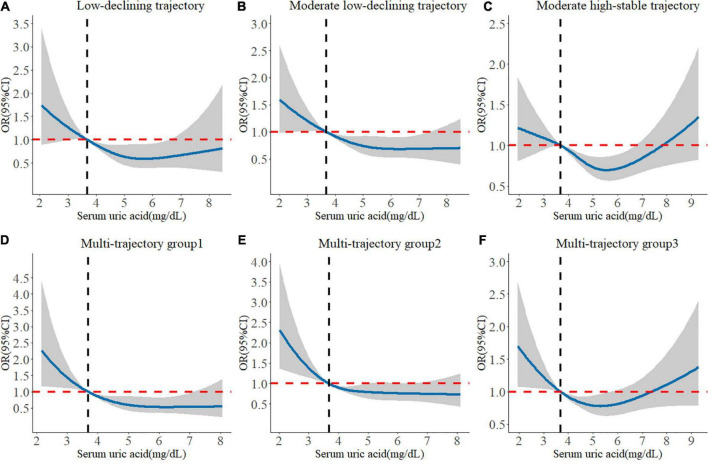
Longitudinal dose-response relationship between baseline SUA levels and poorer cognitive trajectories. The curve was estimated by a restricted cubic spline function with four knots. Solid lines indicate OR. The reference was set to 3.686 mg/dL (25th quantile). The shadow represents 95% confidence intervals. **(A)** Longitudinal dose–response relationship between baseline SUA levels and low-declining global cognitive trajectory. **(B)** Longitudinal dose–response relationship between baseline SUA levels and moderate low-declining global cognitive trajectory. **(C)** Longitudinal dose–response relationship between baseline SUA levels and moderate high-stable global cognitive trajectory. **(D)** Longitudinal dose–response relationship between baseline SUA levels and “episodic memory: low-rapid declining + executive function: low-declining” trajectory. **(E)** Longitudinal dose–response relationship between baseline SUA levels and “episodic memory: low-minimal declining + executive function: moderate-declining” trajectory. **(F)** Longitudinal dose–response relationship between baseline SUA levels and “episodic memory: moderate-stable + executive function: high-declining” trajectory.

We found that higher baseline SUA levels had a lower risk of poorer global cognitive single-trajectories in three adjusted models (Table 2). The odds ratio (OR) of the highest SUA of less optimal trajectories was 0.755 (95% CI: 0.634 - 0.900, *P* for trend = 0.002) as compared with those with the lowest levels of SUA after full adjustment of covariates in model 3. Similarly, higher baseline SUA was negatively associated with worse and declining memory and executive function multi-trajectories. The OR was 0.940 (95% CI: 0.894, 0.990) for SUA per 1 mg/dL increase in model 3. We used RCS to estimate the trend in the risk for each poor trajectory relative to the best trajectory. The spline function for SUA confirmed the non-linear, U-shaped, or J-shaped relationship with the risk of each poor cognitive trajectory. As seen in Figure 2, the protective effect of SUA disappeared when the SUA level was excessively higher.

**TABLE 2:** Associations between baseline SUA and risk of poorer cognitive trajectories.

	Model 1		Model 2		Model 3	
			
	OR (95% CI)	*P*-value	OR (95% CI)	*P*-value	OR (95% CI)	*P*-value
**Global cognitive trajectories**						
SUA, +1 mg/dL	0.907 (0.865, 0.952)	**<0.001**	0.946 (0.899, 0.995)	**0.032**	0.948 (0.901, 0.998)	**0.043**
**By sex-specific quartiles ^a^**						
Q1	Ref.		Ref.		Ref.	
Q2	0.829 (0.703, 0.977)	**0.025**	0.843 (0.710, 1.000)	0.051	0.855 (0.720, 1.016)	0.076
Q3	0.773 (0.656, 0.911)	**0.002**	0.808 (0.680, 0.960)	**0.015**	0.821 (0.690, 0.977)	**0.026**
Q4	0.661 (0.560, 0.780)	**<0.001**	0.747 (0.628, 0.889)	**0.001**	0.755 (0.634, 0.900)	**0.002**
*P* for trend		**<0.001**		**0.001**		**0.002**
**Multi-trajectories**						
SUA, +1 mg/dL	0.904 (0.861, 0.948)	**<0.001**	0.938 (0.892, 0.987)	**0.013**	0.940 (0.894, 0.990)	**0.018**
**By sex-specific quartiles ^a^**						
Q1	Ref.		Ref.		Ref.	
Q2	0.807 (0.684, 0.952)	**0.011**	0.809 (0.681, 0.961)	**0.016**	0.816 (0.687, 0.970)	**0.021**
Q3	0.732 (0.621, 0.864)	**<0.001**	0.748 (0.629, 0.889)	**0.001**	0.761 (0.639, 0.905)	**0.002**
Q4	0.691 (0.585, 0.815)	**<0.001**	0.777 (0.654, 0.924)	**0.004**	0.784 (0.659, 0.933)	**0.006**
*P* for trend		**<0.001**		**0.002**		**0.004**

CI, confidence interval; OR, Odds ratios; SUA, serum uric acid; ref., reference. Model 1: adjusted for age, gender. Model 2: adjusted for age, gender, marital status, education level, smoking status, drinking status, depressive symptoms. Model 3: adjusted for age, gender, marital status, education level, smoking status, drinking status, depressive symptoms, BMI, diabetes, dyslipidemia, stroke, heart-related diseases and prehypertension.

^a^The cutoff values were sex-specific quartiles of SUA (3.399, 4.008, and 4.733 mg/dL for women, 4.188, 4.964, and 5.818 mg/dL for men). Bold P-value denotes statistical significance (*P* < 0.05).

In subgroup analyses based on SUA levels (Table 3), we observed that the negative association of baseline SUA with poorer global cognitive single-trajectories (OR: 0.890; 95% CI: 0.834, 0.950) and worse cognitive multi-trajectories (OR: 0.911; 95% CI: 0.853, 0.973) remained statistically significant among those without hyperuricemia. However, no significant association was found among those with hyperuricemia. Choosing different cut-points for SUA levels to perform other subgroup analyses yielded similar results (sex-specific median or 75th percentile). Notably, among those with ≥ 75th percentile level of SUA, higher SUA levels were positively correlated with poor global cognitive trajectories (OR: 1.048; 95% CI: 0.821, 1.337). Although this result was not statistically significant, it still suggested the adverse effect of higher SUA levels on cognition among those with hyperuricemia.

**TABLE 3:** Associations between baseline SUA and cognitive trajectories in subgroups by SUA levels.

Subgroups	Global cognitive trajectories		Multi-trajectories	
		
	OR (95% CI)	*P*-value	OR (95% CI)	*P*-value
**Cut-points: clinical diagnosis criteria^a^**				
**Non-hyperuricemia group**				
SUA, +1 mg/dL	0.890 (0.834, 0.950)	**<0.001**	0.911 (0.853, 0.973)	**0.006**
Lower-level SUA (by sex-specific median)	Ref.		Ref.	
Higher-level SUA	0.823 (0.721, 0.939)	**0.004**	0.856 (0.748, 0.979)	**0.023**
**Hyperuricemia group**				
SUA, +1 mg/dL	1.169 (0.875, 1.561)	0.289	1.194 (0.884, 1.615)	0.247
Lower-level SUA (by sex-specific median)	Ref.		Ref.	
Higher-level SUA	0.983 (0.531, 1.825)	0.956	1.235 (0.647, 2.366)	0.522
**Cut-points: sex-specific median^b^**				
**Low-level SUA group**				
SUA, +1 mg/dL	0.896 (0.774, 1.038)	0.144	0.838 (0.722, 0.973)	**0.021**
Lower-level SUA (by sex-specific median)	Ref.		Ref.	
Higher-level SUA	0.825 (0.697, 0.978)	**0.026**	0.825 (0.694, 0.981)	**0.029**
**High-level SUA group**				
SUA, +1 mg/dL	0.996 (0.907, 1.092)	0.927	1.005 (0.915, 1.104)	0.913
Lower-level SUA (by sex-specific median)	Ref.		Ref.	
Higher-level SUA	0.902 (0.760, 1.070)	0.238	1.023 (0.859, 1.218)	0.800
**Cut-points: sex-specific 75th quantile^c^**				
**Low-level SUA group**				
SUA, +1 mg/dL	0.901 (0.821, 0.989)	**0.028**	0.870 (0.791, 0.957)	**0.004**
Lower-level SUA (by sex-specific median)	Ref.		Ref.	
Higher-level SUA	0.859 (0.748, 0.987)	**0.032**	0.851 (0.739, 0.980)	**0.025**
**High-level SUA group**				
SUA, +1 mg/dL	1.081 (0.938, 1.245)	0.279	0.983 (0.852, 1.134)	0.815
Lower-level SUA (by sex-specific median)	Ref.		Ref.	
Higher-level SUA	1.048 (0.821, 1.337)	0.707	0.900 (0.704, 1.151)	0.400

CI, confidence interval; OR, Odds ratios; SUA, serum uric acid; ref., reference. Adjusted for: age, gender, marital status, education level, smoking status, drinking status, depressive symptoms, BMI, diabetes, dyslipidemia, stroke, heart-related diseases and prehypertension.

^a^The clinical diagnosis criteria for hyperuricemia were > 6.0 mg/dL for women and > 7.0 mg/dL for men.

^b^The sex-specific median of SUA were 4.008 mg/dL for women, 4.964 mg/dL for men.

^c^The sex-specific 75th percentile of SUA was 4.733 mg/dL for women, 5.818 mg/dL for men. Bold P-value denotes statistical significance (P < 0.05).

### Associations between changes in serum uric acid and 3-year cognitive change

Table 4 shows the association between changes in SUA and future 3-year cognitive change. Compared with participants in the “non-hyperuricemia with elevated SUA” group, participants in the “non-hyperuricemia with decreased SUA” group had a higher risk for decline in global cognitive function (β: -0.387; 95% CI: -0.696, -0.078) and executive function (β: -0.335; 95% CI: -0.555, -0.114). Additionally, the “persistent hyperuricemia” group was negatively associated with improved global cognitive function (β: -0.851; 95% CI: -1.562, -0.140) and enhanced executive function (β: -0.672; 95% CI: -1.180, -0.164). The risk of cognitive decline was higher in the persistent hyperuricemia group. Both the remittent and incident hyperuricemia groups were not significantly associated with cognitive decline.

**TABLE 4:** Associations between changes in SUA and 3-year cognitive changes.

	Global cognitive function		Episodic memory		Executive function	
			
	β (95% CI)	*P*	β (95% CI)	*P*	β (95% CI)	*P*
Non-hyperuricemia with elevated SUA	Ref.		Ref.		Ref.	
Non-hyperuricemia with decreased SUA	–0.387 (–0.696, –0.078)	**0.014**	–0.053 (–0.247, 0.142)	0.595	–0.335 (–0.555, –0.114)	**0.003**
Incident hyperuricemia	0.007 (–0.440, 0.454)	0.976	0.035 (–0.246, 0.317)	0.806	–0.029 (–0.348, 0.291)	0.861
Remittent hyperuricemia	0.055 (–0.789, 0.900)	0.898	–0.074 (–0.606, 0.458)	0.785	0.129 (–0.474, 0.733)	0.674
Persistent hyperuricemia	–0.851 (–1.562, –0.140)	**0.019**	–0.179 (–0.626, 0.269)	0.434	–0.672 (–1.180, –0.164)	**0.010**
**Changes in SUA^a^**						
Changes in SUA, +1 mg/dL	0.075 (–0.049, 0.199)	0.235	0.025 (–0.053, 0.103)	0.528	0.050 (–0.039, 0.138)	0.270
By quartiles^b^						
Q1	Ref.		Ref.		Ref.	
Q2	–0.067 (–0.436, 0.303)	0.723	–0.094 (–0.327, 0.138)	0.426	0.029 (–0.236, 0.293)	0.832
Q3	0.449 (0.073, 0.826)	**0.019**	0.002 (–0.235, 0.239)	0.987	0.447 (0.178, 0.717)	**0.001**
Q4	0.375 (–0.006, 0.757)	0.054	0.089 (–0.152, 0.329)	0.470	0.287 (0.014, 0.560)	**0.040**

CI, confidence interval; β, regression coefficient; SUA, serum uric acid; ref., reference. Adjusted for: age, gender, marital status, education level, smoking status, drinking status, depressive symptoms, BMI, diabetes, dyslipidemia, stroke, heart-related diseases, prehypertension, and SUA levels in 2015.

^a^Changes in SUA was also calculated as the SUA level in 2015 minus that at baseline in 2011.

^b^The cutoff values were quartiles of changes in SUA (−0.073, 0.492, 1.150). Bold P-value denotes statistical significance (P < 0.05).

**FIGURE 3:**
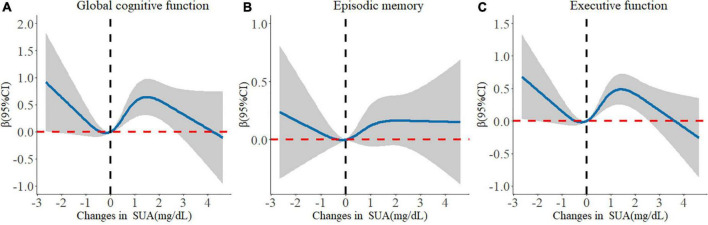
Longitudinal dose-response relationship between changes in serum uric acid and cognitive changes. The curve was estimated by a restricted cubic spline function with four knots. Solid lines indicate the regression coefficient. The reference was set to 0 mg/dL. The shadow represents 95% confidence intervals. **(A)** Longitudinal dose–response relationship between changes in serum uric acid and global cognitive changes. **(B)** Longitudinal dose–response relationship between changes in serum uric acid and episodic memory changes. **(C)** Longitudinal dose–response relationship between changes in serum uric acid and executive function changes.

When the changes in SUA were calculated as the difference of both SUA measurements, only the Q3 group of change was related to improved global cognitive function compared with the Q1 group (β: 0.449; 95% CI: 0.073, 0.826). Moreover, there was a non-linear relationship between changes in SUA and cognitive change. The dose–response relationship indicated that moderate increases in the level of SUA were beneficial to enhance cognitive function (Figure 3).

### Sensitivity analyses

First, we repeated the analyses after further adjusting for eGFR. The results remained almost consistent, as seen in Supplementary Tables 5, 6.

Second, we excluded 2,198 participants without information on physical activity in the first cohort, and the comparison of baseline characteristics between participants included and those who were not included in this analysis is shown in Supplementary Table 7. All variables were not significantly different between groups. The results did not significantly change after further adjusting for physical activity (Supplementary Table 8). Similarly, the SUA levels were significantly negatively associated with worse cognitive trajectories, especially in the non-hyperuricemia group and the low-level SUA group. Conversely, higher SUA levels were significantly positively associated with poorer cognitive trajectories among the hyperuricemia group and the high-level SUA group (75th percentile as cut-points). In the second cohort, 872 participants with assessments of physical activity were included. There was also no significant difference in all variables between the included and excluded populations (Supplementary Table 9). Only the Q4 group of changes in SUA was related to improved global cognitive function compared with the Q1 group (β: 0.629; 95% CI: 0.002, 1.255) (Supplementary Table 10). However, the associations of “non-hyperuricemia with decreased SUA” and “persistent hyperuricemia” with cognitive changes became non-significant, probably due to the reduced sample size.

Moreover, the associations between the SUA levels and cognitive scores over time based on GEE models are shown in Supplementary Table 11. With each 1 mg/dl increase of SUA, the global cognitive score increased by 0.071 (95%CI: 0.015, 0.128) and the executive function score increased by 0.077 (95%CI: 0.035, 0.118). Similarly, a protective role for SUA was only found among those with normal or lower SUA. Interestingly, it seems that higher SUA levels could significantly impair episodic memory function among those with hyperuricemia [clinical diagnosis criteria for hyperuricemia (Huang Y. et al., 2022), sex-specific median, and 75th percentile as cut-points].

In addition, the results of subgroup analyses are presented in Supplementary Figure 4. The protective effects of SUA on better cognitive trajectories were significant in all subgroups by age, depression status, and the presence of prehypertension. Such association was observed among women but not among men. When non-normotensive patients were complicated by at least 1 CMD, the protective effect of SUA also disappeared.

Finally, the results were similar when the cognitive score in 2018 was used as the secondary outcome, which are shown in Supplementary Table 12.

## Discussion

In this cohort study, four single-trajectories of global cognitive performance and four multi-trajectories of executive function and episodic memory were identified in prehypertensive and hypertensive populations. We demonstrated that higher baseline SUA levels were negatively associated with a declining and poorer 7-year cognitive performance trajectory in a non-normotensive population. In subgroup analyses, the associations only existed among those with normal or lower levels of SUA. Reciprocally, higher baseline SUA may impair cognitive function among patients with hyperuricemia. Additionally, 4-year changes in SUA could influence future 3-year cognitive changes. Maintenance of the normal SUA levels and decreased levels of SUA at the same time can disrupt cognitive function. For patients with hyperuricemia in 2011, when SUA levels decreased to the normal range in 2015, no adverse effects on cognitive performance were found. Reversely, the persistent presence of hyperuricemia increased the risk of cognitive decline. In summary, our findings confirm the neuroprotective effect of SUA. However, under only the normal levels of SUA, moderate increases in SUA can prevent a decline in cognitive function among the non-normotensive population.

Based on our results, 27.19% of individuals with prehypertension and hypertension had a significant trend of decline in global cognitive function. Meanwhile, 31.04% of non-normotensive populations had a marked decreasing trend in both episodic memory and executive function. Non-normotensive patients had a high risk of incident cognitive decline. Then, we found that the non-normotensive population with high levels of SUA still had more favorable 7-year cognitive trajectories than those with low levels of SUA, which is similar to previous studies in the general population (Wang T. et al., 2017; Xiu et al., 2017; Scheepers et al., 2019; Chen et al., 2021). One potential protection mechanism may be the antioxidant effects of SUA (Tana et al., 2018). Oxidative stress or low antioxidant levels in the brain may exert an important role in the pathogenesis of cognitive impairment or dementia, leading to free radical generation, lipid peroxidation, and mitochondrial dysfunction (Zhou et al., 2021). While higher SUA could protect the blood-brain barrier against oxidative stress (Tana et al., 2018). Moreover, patients with congenital SUA disorder may have a genetic tendency to cognitive dysfunction (Qiao et al., 2021). Then, hypouricemia is often accompanied by poor nutritional status, which results in cognitive impairment (Sanders et al., 2016; Zhou et al., 2021). Thus, people with higher SUA levels would show better cognitive performance.

However, the results of some previous studies are contradictory. A study reported that higher levels of SUA were associated with faster cognitive decline (Alam et al., 2020). Another finding of a 12-year cohort study demonstrated that high SUA levels may increase the risk of dementia, especially vascular or mixed dementia (Latourte et al., 2018b). This inconsistency could be explained by the included patient with hyperuricemia. Cardiometabolic disorders, inflammation, vascular damage, and the pro-arteriosclerotic effect caused by higher SUA levels may counteract its neuroprotective role, resulting in cognitive decline and increasing the risk of vascular dementia (Tana et al., 2018; Zhou et al., 2021; Huang Y. et al., 2022). Thus, we performed several subgroup analyses with different SUA levels. We found that the non-normotensive population with higher SUA levels has more stable and better cognitive trajectories only in non-hyperuricemia groups, which is similar to one previous study (Chen et al., 2021). The sample size of adults with hyperuricemia in both our study and of the study of Chen et al. (2021) was relatively small. Considering this limitation, subgroups were divided by different cut-points for SUA levels (sex-specific median and 75th percentile) and similar results were obtained. The significant protective effect of SUA on cognitive function was found only in low-level SUA subgroups. Notably, among those with higher or abnormal levels of SUA, SUA could impair their cognitive function, especially memory function. The optimum cut-points of the abnormal levels of SUA to balance the integrated prevention of cognitive impairment and cardiovascular and cerebrovascular diseases should be further explored. Besides, the dose–response relationship test further confirmed our results.

In sensitivity analyses, the protective effect of SUA also disappeared when non-normotensive patients were complicated by at least 1 CMD, which could further verify our conjecture mentioned above. Additionally, the finding of this association only in women, rather than in men, may be explained by the higher risk of cerebrovascular disease in men and the estrogen effects after menopause in women (Lee et al., 2021). Some studies reported similar research conclusions (Scheepers et al., 2019; Lee et al., 2021). Our findings suggested that the neuroprotective role of SUA should be emphasized in female patients with hypertension without other CMD.

Innovatively, we also provide evidence that the changes in SUA can influence future cognitive performance. When SUA was maintained at normal levels over a period of time, decreased levels of SUA are strongly associated with worse cognitive performance. However, the persistent presence of hyperuricemia may result in more severe cognitive decline. Additionally, we analyzed the changes in SUA as a continuous variable and the dose–response relationship. We proved that a moderate increase in SUA levels may improve cognitive function, rather than an excessive increase. When SUA increased to abnormal levels, the protective effect of SUA may disappear. SUA appears to be a double-edged sword due to both the harmful effect on patients with hypertension and the neuroprotective role (Latourte et al., 2018a; Yang et al., 2021). In clinical practice, it also remains unknown whether urate-lowering therapy has adverse effects on cognitive performance, which is of great concern (Singh, 2018; Latourte et al., 2021). To a certain extent, our study could provide supporting evidence that remittent hyperuricemia was not associated with cognitive decline. Conversely, the risk for cognitive decline may be even higher if left untreated or if higher SUA levels are not controlled. Decreased SUA may result in poorer cognitive changes only in those without hyperuricemia. Our findings provide some inspiration for balancing the urate-lowering therapy against the prevention of cognitive impairment or dementia. The maintenance of normal SUA levels is a primary condition to prevent cognitive impairment and dementia. Thus, the treatment of hyperuricemia should still be a priority in daily life and clinical practice for non-normotensive populations.

### Strengths and limitations

First, our findings extend and refine evidence that higher SUA levels within the normal range could still protect cognitive functions in the prehypertensive and hypertensive populations. Additionally, single-time point measures of SUA and changes in SUA over time were all considered. Furthermore, our study fully considered different situations among both patients with non-hyperuricemia and hyperuricemia, which provides specific guidance to balance urate-lowering therapy against the prevention of cognitive impairment or dementia. The other strength is the use of GBTM and GBMTM to identify groups of individuals who experienced similar levels and change patterns of cognitive functions over time (Li et al., 2020), while the traditional statistical approaches for repeated measures data, such as GEE or linear mixed models, only focus on mean population trajectories (Li et al., 2020). However, the conventional method, GEE, was also used to validate the robustness of our results. Finally, the strengths of our study also include the prospective cohort design and the use of a large nationwide representative sample covering 28 provinces in China.

We acknowledge several limitations of this study. First, the physical activity investigation was limited to a randomly selected subgroup in the CHARLS study and had a large number of missing values. In the sensitivity analysis, physical activity was adjusted for only in the subpopulation with a small sample size. Second, residual confounding factors were not fully controlled. Dietary intake, nutrients, and urate-lowering medications were also not incorporated in the final model due to the lack of related data. Additionally, the assessment tool for cognition performance used was relatively simple and limited. The cutoff values of cognitive impairment of this test were lacking, but the moderate-to-high validity of this cognitive test was reported compared with the Mini-Mental State Examination (MMSE) and the Clinical Dementia Rating Scale (CDR) (Meng et al., 2019). Finally, more time point measurements of the SUA levels need to be conducted to capture the longitudinal patterns of SUA over a period more accurately.

## Conclusion

In brief, our findings indicate that baseline SUA and changes in SUA can affect future cognitive trajectories or changes in a non-normotensive population. The maintenance of normal SUA levels and a moderate increase of SUA may be beneficial for enhanced cognitive function. However, the persistent presence of hyperuricemia may result in cognitive decline. Although the neuroprotective role of high levels of SUA was confirmed in our study, the maintenance of normal SUA levels is a primary condition to prevent cognitive impairment and dementia.

## Data availability statement

The raw data supporting the conclusions of this article will be made available by the authors, without undue reservation.

## Ethics statement

The studies involving human participants were reviewed and approved by Ethical approval for all the CHARLS waves was granted from the Institutional Review Board at Peking University. The IRB approval number for the main household survey, including anthropometrics, is IRB00001052-11015. The patients/participants provided their written informed consent to participate in this study.

## Author contributions

LT: full access to all of the data in the study and takes responsibility for the integrity of the data and the accuracy of the data analysis. JW, XG, and LT: study conception and design. JW, RJ, ZH, ZX, and LT: data collection. JW, ZW, YL, YJL, XJ, and LT: data analysis and interpretation. JW and LT: manuscript writing and reviewing. XG and LT: study supervision. All authors read and approved the final manuscript.

## Abbreviations

SUA: serum uric acid
GBTM: group-based trajectory model
CHARLS: China Health and Retirement Longitudinal Study
BMI: body mass index
eGFR: estimated glomerular filtration rate
CMD: cardiometabolic diseases
SBP: systolic blood pressure
DBP: diastolic blood pressure
SD: standard deviation
GBMTM: group-based multi-trajectory modeling
BIC: Bayesian information criteria
AIC: Akaike information criterion
APP: average posterior probabilities
CI: confidence interval
RCS: restricted cubic spline
GEE: generalized estimating equation
OR: odds ratio.
