# Supplementary material for: Moderate increase of serum uric acid within a normal range is associated with improved cognitive function in a non-normotensive population: A nationally representative cohort study
Source: Front Aging Neurosci. 2022 Sep 1;14:944341. doi: 10.3389/fnagi.2022.944341 (PMC9475062; doi:10.3389/fnagi.2022.944341)
Supplement: Supplementary file 1 [file Data_Sheet_1.pdf]

Supplemental Content

Table S1. Fit statistics for global cognitive trajectories in a non-normotensive population from CHARLS
Table S2. Fit statistics for cognitive multi-trajectories in a non-normotensive population from CHARLS
Table S3. The final four-group trajectory model of global cognitive scores
Table S4. The final four-group trajectory model of episodic memory and executive function
Table S5. Associations between baseline SUA and cognitive trajectories after further adjusting for eGFR
Table S6. Associations between changes in SUA and 3-year cognitive changes after further adjusting for eGFR
Table S7. Baseline characteristics between participants with assessments of physical activity (included) and without assessments of physical activity (excluded) in the first cohort
Table S8. Associations between baseline SUA and cognitive trajectories in subpopulations of 1707 participants with assessments of physical activity
Table S9. Baseline characteristics between participants with assessments of physical activity (included) and without assessments of physical activity (excluded) in the second cohort
Table S10. Associations between changes in SUA and 3-year cognitive changes in subpopulations of 872 participants with assessments of physical activity
Table S11. Associations between SUA and longitudinal cognition according to generalized estimating equations
Table S12. Associations between changes in SUA and cognitive scores in 2018
Figure S1. Study flowchart of participant selection in the first cohort
Figure S2. Study flowchart of participant selection in the second cohort
Figure S3. The time line of the study
Figure S4. Associations between baseline SUA and cognitive trajectories in different subgroups

Table S1. Fit statistics for global cognitive trajectories in a non-normotensive population from CHARLS

Fit statistic	Number of classes				
	2	3	4	5	6
BIC [a]	-34456.42	-33856.20	-33647.51	-33617.60	-33582.86
AIC [a]	-34437.61	-33861.82	-33609.89	-33570.58	-33526.43
Class proportion [b]	Class1, 27.98%	Class1, 13.34%	Class1, 7.50%	Class1, 5.80%	Class1, 5.16%
	Class2, 72.02%	Class2, 53.99%	Class2, 19.69%	Class2, 18.61%	Class2, 10.25%
		Class3, 32.66%	Class3, 37.70%	Class3, 14.59%	Class3, 23.63%
			Class4, 35.11%	Class4, 22.15%	Class4, 10.56%
				Class5, 38.85%	Class5, 11.95%
					Class6, 38.46%
APP [c]	Class1, 0.94	Class1, 0.92	Class1, 0.89	Class1, 0.89	Class1, 0.88
	Class2, 0.97	Class2, 0.93	Class2, 0.85	Class2, 0.77	Class2, 0.63
		Class3, 0.86	Class3, 0.81	Class3, 0.81	Class3, 0.81
			Class4, 0.86	Class4, 0.73	Class4, 0.65
				Class5, 0.76	Class5, 0.76
					Class6, 0.76

Abbreviations: BIC, Bayesian Information Criterion; AIC, Akaike Information; APP, average posterior probabilities; CHARLS, China Health and Retirement Longitudinal Study

[a] A lower absolute value suggests a better model fit.

[b] No less than 5% of total count in a class.

[c] A higher value is better (preferably > 0.70 in a class).

Table S2. Fit statistics for cognitive multi-trajectories in a non-normotensive population from CHARLS

Fit statistic	Number of classes				
	2	3	4	5	6
BIC [a]	-55568.40	-54683.17	-54225.90	-54051.88	-53908.69
AIC [a]	-55533.91	-54633.01	-54160.06	-53970.37	-53811.51
Class proportion [b]	Class1, 26.35%	Class1, 12.07%	Class1, 8.45%	Class1, 8.93%	Class1, 5.19%
	Class2, 73.65%	Class2, 29.77%	Class2, 22.59%	Class2, 15.41%	Class2, 13.40%
		Class3, 58.16%	Class3, 44.25%	Class3, 43.72%	Class3, 16.78%
			Class4, 24.71%	Class4, 9.04%	Class4, 40.51%
				Class5, 22.91%	Class5, 13.08%
					Class6, 11.03%
APP [c]	Class1, 0.94	Class1, 0.93	Class1, 0.92	Class1, 0.91	Class1, 0.90
	Class2, 0.98	Class2, 0.88	Class2, 0.88	Class2, 0.81	Class2, 0.74
		Class3, 0.95	Class3, 0.84	Class3, 0.85	Class3, 0.83
			Class4, 0.85	Class4, 0.79	Class4, 0.83
				Class5, 0.85	Class5, 0.84
					Class6, 0.76

Abbreviations: BIC, Bayesian Information Criterion; AIC, Akaike Information; APP, average posterior probabilities; CHARLS, China Health and Retirement Longitudinal Study

[a] A lower absolute value suggests a better model fit.

[b] No less than 5% of total count in a class.

[c] A higher value is better (preferably > 0.70 in a class).

Table S3. The final four-group trajectory model of global cognitive scores

Trajectory group	Maximum likelihood estimates				
	**Parameter**	**Est**	**SE**	**Z value**	***P***
**Class1: Low-declining, 7.50%**	Intercept	957.80	69.31	13.82	<0.001
	Linear	-0.47	0.03	-13.75	<0.001
**Class2: moderate low-declining, 19.69%**	Intercept	731.16	46.29	15.80	<0.001
	Linear	-0.36	0.02	-15.60	<0.001
**Class3: moderate high-stable, 37.70%**	Intercept	223.19	32.28	6.92	<0.001
	Linear	-0.10	0.02	-6.53	<0.001
**Class4: high-stable, 35.11%**	Intercept	11.37	28.21	0.40	0.69
	Linear	0.001	0.01	0.13	0.90

Abbreviations: Est, parameter estimate; SE, standard error of parameter estimate.

Table S4. The final four-group trajectory model of episodic memory and executive function

Trajectory group	Maximum likelihood estimates					
	**Variables**	**Parameter**	**Est**	**SE**	**Z value**	**P**
**Class1, 8.45%**	Episodic memory	Intercept	505.03	40.71	12.405	<0.001
Episodic memory: low-rapid declining		Linear	-0.25	0.02	-12.358	<0.001
Executive function: low-declining	Executive function	Intercept	536.29	48.57	11.04	<0.001
		Linear	-0.26	0.02	-10.98	<0.001
**Class2, 22.59%**	Episodic memory	Intercept	192.13	25.78	7.453	<0.001
Episodic memory: low- minimal declining		Linear	-0.09	0.01	-7.343	<0.001
Executive function: moderate-declining	Executive function	Intercept	472.56	30.45	15.52	<0.001
		Linear	-0.23	0.02	-15.30	<0.001
**Class3, 44.25%**	Episodic memory	Intercept	-4.87	18.19	-0.27	0.79
Episodic memory: moderate-stable		Linear	0.004	0.01	0.46	0.64
Executive function: high-declining	Executive function	Intercept	181.07	20.10	9.01	<0.001
		Linear	-0.09	0.01	-8.55	<0.001
**Class4, 24.71%**	Episodic memory	Intercept	-126.15	21.36	-5.91	<0.001
Episodic memory: high–rising		Linear	0.07	0.01	6.15	<0.001
Executive function: high-stable	Executive function	Intercept	124.92	23.87	5.23	<0.001
		Linear	-0.06	0.01	-4.81	<0.001

Abbreviations: Est, parameter estimate; SE, standard error of parameter estimate.

Table S5. Associations between baseline SUA and cognitive trajectories after further adjusting for eGFR

Subgroups	Global cognitive trajectories		Multi-trajectories	
	OR (95% CI)	*P* value	OR (95% CI)	*P* value
**All subjects**				
SUA, +1 mg/dL	0.951 (0.899, 1.004)	0.071	0.951 (0.901, 1.005)	0.073
By sex-specific quartiles				
Q1	Ref.		Ref.	
Q2	0.858 (0.720, 1.022)	0.086	0.836 (0.701, 0.995)	**0.044**
Q3	0.821 (0.686, 0.981)	**0.030**	0.778 (0.651, 0.931)	**0.006**
Q4	0.755 (0.627, 0.910)	**0.003**	0.813 (0.676, 0.977)	**0.028**
**Subgroups by SUA levels**				
**Cut-points: clinical diagnosis criteria [a]**				
**Non-hyperuricemia group**				
SUA, +1 mg/dL	0.895 (0.835, 0.958)	**0.001**	0.922 (0.860, 0.988)	**0.022**
Lower-level SUA (by sex-specific median)	Ref.		Ref.	
Higher-level SUA	0.839 (0.731, 0.962)	**0.012**	0.883 (0.768, 1.015)	0.080
**Hyperuricemia group**				
SUA, +1 mg/dL	1.165 (0.864, 1.569)	0.315	1.198 (0.879, 1.636)	0.253
Lower-level SUA (by sex-specific median)	Ref.		Ref.	
Higher-level SUA	0.948 (0.505, 1.785)	0.869	1.217 (0.629, 2.361)	0.560
**Cut-points: sex-specific median**				
**Low-level SUA group**				
SUA, +1 mg/dL	0.921 (0.791, 1.072)	0.288	0.858 (0.735, 1.001)	0.051
Lower-level SUA (by sex-specific median)	Ref.		Ref.	
Higher-level SUA	0.847 (0.712, 1.008)	0.062	0.849 (0.711, 1.013)	0.070
**High-level SUA group**				
SUA, +1 mg/dL	0.985 (0.895, 1.083)	0.749	1.008 (0.915, 1.109)	0.878
Lower-level SUA (by sex-specific median)	Ref.		Ref.	
Higher-level SUA	0.892 (0.750, 1.061)	0.199	1.029 (0.863, 1.228)	0.749
**Cut-points: sex-specific 75th quantile**				
**Low-level SUA group**				
SUA, +1 mg/dL	0.915 (0.830, 1.009)	0.076	0.879 (0.796, 0.972)	**0.012**
Lower-level SUA (by sex-specific median)	Ref.		Ref.	
Higher-level SUA	0.829 (0.709, 0.969)	**0.018**	0.808 (0.688, 0.948)	**0.009**
**High-level SUA group**				
SUA, +1 mg/dL	1.060 (0.917, 1.225)	0.426	0.99 (0.856, 1.145)	0.894
Lower-level SUA (by sex-specific median)	Ref.		Ref.	
Higher-level SUA	1.269 (0.931, 1.732)	0.133	1.039 (0.765, 1.411)	0.805

Abbreviations: CI, confidence interval; OR, Odds ratios; eGFR, estimated glomerular filtration rate; SUA, serum uric acid;

ref., reference.
Adjusted for: age, gender, marital status, education level, smoking status, drinking status, depressive symptoms, BMI, eGFR, diabetes, dyslipidemia, stroke, heart-related diseases and prehypertension.
[a] The clinical diagnosis criteria for hyperuricemia were >6.0 mg/dL for women and >7.0 mg/dL for men

Table S6. Associations between changes in SUA and 3-year cognitive changes after further adjusting for eGFR

	Global cognitive function		Episodic memory		Executive function	
	***β* (95% CI)**	***P***	***β* (95% CI)**	***P***	***β* (95% CI)**	***P***
Non-hyperuricemia with elevated SUA	Ref.		Ref.		Ref.	
Non-hyperuricemia with decreased SUA	-0.367 (-0.677, -0.056)	**0.021**	-0.044 (-0.239, 0.151)	0.659	-0.323 (-0.544, -0.101)	**0.004**
Incident hyperuricemia	0.049 (-0.402, 0.500)	0.830	0.053 (-0.231, 0.337)	0.714	-0.004 (-0.326, 0.319)	0.982
Remittent hyperuricemia	0.137 (-0.714, 0.988)	0.752	-0.040 (-0.576, 0.496)	0.884	0.177 (-0.431, 0.786)	0.568
Persistent hyperuricemia	-0.746 (-1.468, -0.023)	**0.043**	-0.135 (-0.590, 0.320)	0.562	-0.611 (-1.128, -0.095)	**0.020**
**Changes in SUA** [a]						
Changes in SUA, +1 mg/dL	0.076 (-0.048, 0.200)	0.229	0.025 (-0.053, 0.103)	0.525	0.051 (-0.038, 0.139)	0.261
By quartiles [b]						
Q1	Ref.		Ref.		Ref.	
Q2	-0.059 (-0.428, 0.311)	0.756	-0.092 (-0.324, 0.141)	0.441	0.033 (-0.231, 0.297)	0.807
Q3	0.456 (0.079, 0.832)	**0.018**	0.004 (-0.234, 0.241)	0.975	0.452 (0.182, 0.721)	**0.001**
Q4	0.377 (-0.004, 0.759)	0.053	0.089 (-0.151, 0.330)	0.467	0.288 (0.015, 0.561)	**0.039**

Abbreviations: CI, confidence interval; *β*, regression coefficient; eGFR, estimated glomerular filtration rate; SUA, serum uric acid.; ref., reference.

Adjusted for: age, gender, marital status, education level, smoking status, drinking status, depressive symptoms, BMI, diabetes, dyslipidemia, stroke, heart-related diseases, prehypertension, eGFR, and SUA levels in 2015.

[a] Changes in SUA was also calculated as the SUA level in 2015 minus that at baseline in 2011.

[b] The cutoff values were quartiles of changes in SUA (-0.073, 0.492, 1.150).

Table S7. Baseline characteristics between participants with assessments of physical activity (included) and without assessments of physical activity (excluded) in the first cohort

Characteristics	Overall	Included	Excluded	*P* value
**No. of participants**	3905	1707	2198	
**Age(years), mean (SD)**	58.48 (8.54)	58.36 (8.43)	58.57 (8.63)	0.436
**Male, n (%)**	1968 (50.4)	853 (50.0)	1115 (50.7)	0.662
**Married, n (%)**	3383 (86.6)	1476 (86.5)	1907 (86.8)	0.826
**Educational level, n (%)**				0.949
No formal education	1400 (35.9)	616 (36.1)	784 (35.7)	
Junior high school or below	1997 (51.1)	868 (50.8)	1129 (51.4)	
High school or above	508 (13.0)	223 (13.1)	285 (13.0)	
**Smoking status, n (%)**				0.270
Non-smoker	2309 (59.1)	1034 (60.6)	1275 (58.0)	
Former smoker	391 (10.0)	165 (9.7)	226 (10.3)	
Current smoker	1205 (30.9)	508 (29.8)	697 (31.7)	
**Drinking status, n (%)**				0.380
More than once a month	1072 (27.5)	463 (27.1)	609 (27.7)	
Less than once a month	312 (8.0)	126 (7.4)	186 (8.5)	
None of these	2521 (64.6)	1118 (65.5)	1403 (63.8)	
**Depressive symptoms, n (%)**	875 (22.4)	401 (23.5)	474 (21.6)	0.163
**BMI (kg/m^2), n (%)**				0.288
<18.5	160 (4.1)	68 (4.0)	92 (4.2)	
18.5-23.9	1829 (46.8)	777 (45.5)	1052 (47.9)	
≥24.0	1916 (49.1)	862 (50.5)	1054 (48.0)	
**CMD, n (%)**				
Diabetes	295 (7.6)	123 (7.2)	172 (7.8)	0.506
Dyslipidemia	510 (13.1)	230 (13.5)	280 (12.7)	0.530
Stroke	76 (1.9)	37 (2.2)	39 (1.8)	0.444
Heart-related diseases	559 (14.3)	247 (14.5)	312 (14.2)	0.843
**At least 1 CMD, n (%)**	1080 (27.7)	475 (27.8)	605 (27.5)	0.863
**Prehypertension, n (%)**	1596 (40.9)	704 (41.2)	892 (40.6)	0.702
**eGFR (mL/min/1.73 m^2), mean (SD)**	91.97 (14.86)	92.16 (14.22)	91.82 (15.35)	0.477
**SUA (mg/dL), mean (SD)**	4.61 (1.29)	4.56 (1.28)	4.64 (1.30)	0.067
**Cognitive scores, mean (SD)**				
Global cognitive function	12.23 (3.50)	12.18 (3.46)	12.26 (3.52)	0.453
Executive function	8.45 (2.55)	8.41 (2.54)	8.48 (2.55)	0.402
Episodic memory	3.78 (1.67)	3.77 (1.65)	3.78 (1.68)	0.772

Data are presented as the mean (SD), median [IQR] or number (%), as appropriate. Continuous variables were compared using one-way ANOVA test or Kruskal-Wallis test. Categorical variables were compared using χ^2 test or Fisher's exact test. Abbreviations: BMI, body mass index; CMD, cardiometabolic diseases; eGFR, estimated glomerular filtration rate; SUA, serum uric acid.

Table S8. Associations between baseline SUA and cognitive trajectories in subpopulations of 1707 participants with assessments of physical activity

Subgroups	Global cognitive trajectories		Multi-trajectories	
	**OR (95% CI)**	***P* value**	**OR (95% CI)**	***P* value**
**All subjects**				
SUA, +1 mg/dL	0.947 (0.876, 1.024)	0.177	0.910 (0.842, 0.984)	**0.018**
By sex-specific quartiles				
Q1	Ref.		Ref.	
Q2	0.782 (0.601, 1.017)	0.067	0.732 (0.561, 0.955)	**0.022**
Q3	0.688 (0.525, 0.900)	**0.006**	0.670 (0.511, 0.878)	**0.004**
Q4	0.735 (0.553, 0.976)	**0.034**	0.694 (0.522, 0.920)	**0.011**
**Subgroups by SUA levels**				
**Cut-points: clinical diagnosis criteria [a]**				
**Non-hyperuricemia group**				
SUA, +1 mg/dL	0.863 (0.781, 0.952)	**0.003**	0.891 (0.806, 0.985)	**0.024**
Lower-level SUA (by sex-specific median)	Ref.		Ref.	
Higher-level SUA	0.766 (0.628, 0.935)	**0.009**	0.808 (0.66, 0.989)	**0.039**
**Hyperuricemia group**				
SUA, +1 mg/dL	1.673 (1.080, 2.650)	**0.023**	1.286 (0.825, 2.019)	0.269
Lower-level SUA (by sex-specific median)	Ref.		Ref.	
Higher-level SUA	3.730 (0.996, 14.601)	0.053	1.822 (0.479, 7.152)	0.382
**Cut-points: sex-specific median**				
**Low-level SUA group**				
SUA, +1 mg/dL	1.003 (0.802, 1.253)	0.982	0.925 (0.738, 1.160)	0.501
Lower-level SUA (by sex-specific median)	Ref.		Ref.	
Higher-level SUA	0.931 (0.721, 1.203)	0.585	0.859 (0.661, 1.115)	0.253
**High-level SUA group**				
SUA, +1 mg/dL	1.055 (0.917, 1.214)	0.451	0.973 (0.842, 1.124)	0.709
Lower-level SUA (by sex-specific median)	Ref.		Ref.	
Higher-level SUA	0.871 (0.669, 1.132)	0.302	0.956 (0.731, 1.250)	0.741
**Cut-points: sex-specific 75th quantile**				
**Low-level SUA group**				
SUA, +1 mg/dL	0.909 (0.79, 1.046)	0.184	0.885 (0.768, 1.020)	0.092
Lower-level SUA (by sex-specific median)	Ref.		Ref.	
Higher-level SUA	0.778 (0.619, 0.979)	**0.032**	0.736 (0.583, 0.929)	**0.010**
**High-level SUA group**				
SUA, +1 mg/dL	1.271 (1.022, 1.584)	**0.031**	0.935 (0.746, 1.169)	0.556
Lower-level SUA (by sex-specific median)	Ref.		Ref.	
Higher-level SUA	2.219 (1.361, 3.647)	**0.002**	1.076 (0.657, 1.760)	0.772

Abbreviations: CI, confidence interval; OR, Odds ratios; SUA, serum uric acid.; ref., reference.

Adjusted for: age, gender, marital status, education level, smoking status, drinking status, depressive symptoms, BMI, physical activity, diabetes, dyslipidemia, stroke, heart-related diseases and prehypertension.

[a] The clinical diagnosis criteria for hyperuricemia were >6.0 mg/dL for women and >7.0 mg/dL for men

Table S9. Baseline characteristics between participants with assessments of physical activity (included) and without assessments of physical activity (excluded) in the second cohort

Characteristics	Overall	Included	Excluded	*P* value
**No. of participants**	2077	872	1205	
**Age(years), mean (SD)**	57.78 (8.06)	57.76 (8.09)	57.79 (8.04)	0.927
**Male, n (%)**	1046 (50.4)	423 (48.5)	623 (51.7)	0.164
**Married, n (%)**	1833 (88.3)	760 (87.2)	1073 (89.0)	0.211
**Educational level, n (%)**				0.996
No formal education	654 (31.5)	275 (31.5)	379 (31.5)	
Junior high school or below	1136 (54.7)	476 (54.6)	660 (54.8)	
High school or above	287 (13.8)	121 (13.9)	166 (13.8)	
**Smoking status, n (%)**				0.323
Non-smoker	1243 (59.8)	537 (61.6)	706 (58.6)	
Former smoker	206 (9.9)	79 (9.1)	127 (10.5)	
Current smoker	628 (30.2)	256 (29.4)	372 (30.9)	
**Drinking status, n (%)**				0.682
More than once a month	569 (27.4)	233 (26.7)	336 (27.9)	
Less than once a month	177 (8.5)	71 (8.1)	106 (8.8)	
None of these	1331 (64.1)	568 (65.1)	763 (63.3)	
**Depressive symptoms, n (%)**	504 (24.3)	213 (24.4)	291 (24.1)	0.925
**BMI (kg/m^2), n (%)**				0.351
<18.5	81 (3.9)	31 (3.6)	50 (4.1)	
18.5-23.9	897 (43.2)	364 (41.7)	533 (44.2)	
≥24.0	1099 (52.9)	477 (54.7)	622 (51.6)	
**CMD, n (%)**				
Diabetes	154 (7.4)	68 (7.8)	86 (7.1)	0.629
Dyslipidemia	273 (13.1)	112 (12.8)	161 (13.4)	0.781
Stroke	31 (1.5)	18 (2.1)	13 (1.1)	0.100
Heart-related diseases	295 (14.2)	121 (13.9)	174 (14.4)	0.765
**At least 1 CMD, n (%)**	573 (27.6)	243 (27.9)	330 (27.4)	0.847
**Prehypertension, n (%)**	1011 (48.7)	432 (49.5)	579 (48.0)	0.531
**eGFR (mL/min/1.73 m^2), mean (SD)**	92.77 (14.43)	92.57 (14.51)	92.92 (14.37)	0.588
**SUA (mg/dL), mean (SD)**	5.11 (1.41)	5.08 (1.39)	5.13 (1.43)	0.417
**Cognitive scores, mean (SD)**				
Global cognitive function	12.32 (3.57)	12.27 (3.56)	12.36 (3.58)	0.567
Executive function	8.54 (2.56)	8.47 (2.55)	8.58 (2.57)	0.315
Episodic memory	3.79 (1.67)	3.80 (1.69)	3.78 (1.65)	0.752
**Changes in SUA**				0.215
No hyperuricemia & increased SUA	1258 (60.6)	553 (63.4)	705 (58.5)	
No hyperuricemia & decreased SUA	499 (24.0)	194 (22.2)	305 (25.3)	
Incident hyperuricemia	199 (9.6)	77 (8.8)	122 (10.1)	
Remittent hyperuricemia	50 (2.4)	22 (2.5)	28 (2.3)	
Persistent hyperuricemia	71 (3.4)	26 (3.0)	45 (3.7)	
**Changes in SUA (mg/dL), mean (SD)**	0.57 (1.10)	0.61 (1.10)	0.54 (1.10)	0.168

Data are presented as the mean (SD), median [IQR] or number (%), as appropriate. Continuous variables were compared using one-way ANOVA test or Kruskal-Wallis test. Categorical variables were compared using χ^2 test or Fisher's exact test.

Abbreviations: BMI, body mass index; CMD, cardiometabolic diseases; eGFR, estimated glomerular filtration rate; SUA, serum uric acid.

Table S10. Associations between changes in SUA and 3-year cognitive changes in subpopulations of 872 participants with assessments of physical activity

	Global cognitive function		Episodic memory		Executive function	
	***β* (95% CI)**	***P***	***β* (95% CI)**	***P***	***β* (95% CI)**	***P***
Non-hyperuricemia with elevated SUA	Ref.		Ref.		Ref.	
Non-hyperuricemia with decreased SUA	-0.286 (-0.804, 0.232)	0.279	-0.153 (-0.481, 0.174)	0.358	-0.133 (-0.499, 0.234)	0.478
Incident hyperuricemia	0.013 (-0.747, 0.773)	0.973	-0.066 (-0.546, 0.414)	0.788	0.079 (-0.458, 0.616)	0.773
Remittent hyperuricemia	-0.315 (-1.690, 1.060)	0.653	-0.312 (-1.181, 0.557)	0.481	-0.003 (-0.975, 0.969)	0.995
Persistent hyperuricemia	-0.585 (-1.833, 0.663)	0.358	0.123 (-0.665, 0.911)	0.760	-0.708 (-1.590, 0.174)	0.116
**Changes in SUA [a]**						
Changes in SUA, +1 mg/dL	0.139 (-0.064, 0.342)	0.179	0.108 (-0.020, 0.236)	0.099	0.031 (-0.112, 0.175)	0.667
By quartiles [b]						
Q1	Ref.		Ref.		Ref.	
Q2	-0.351 (-0.956, 0.254)	0.256	-0.090 (-0.473, 0.293)	0.645	-0.247 (-0.677, 0.183)	0.260
Q3	0.223 (-0.398, 0.844)	0.481	0.046 (-0.347, 0.439)	0.820	0.172 (-0.268, 0.613)	0.443
Q4	0.629 (0.002, 1.255)	**0.049**	0.366 (-0.031, 0.763)	0.070	0.258 (-0.186, 0.703)	0.254

Abbreviations: CI, confidence interval; *β*, regression coefficient; SUA, serum uric acid.; ref., reference.

Adjusted for: age, gender, marital status, education level, smoking status, drinking status, depressive symptoms, BMI, diabetes, dyslipidemia, stroke, heart-related diseases, prehypertension, physical exercise, and SUA levels in 2015.

[a] Changes in SUA was also calculated as the SUA level in 2015 minus that at baseline in 2011.

[b] The cutoff values were quartiles of changes in SUA (-0.041, 0.533, 1.199).

Table S11. Associations between SUA and longitudinal cognition according to generalized estimating equations

Subgroups	Global cognitive function		Episodic memory		Executive function	
	β (95% CI)	*P*	β (95% CI)	*P*	β (95% CI)	*P*
**All subjects**						
SUA, +1 mg/dL	0.071 (0.015, 0.128)	**0.013**	-0.005 (-0.033, 0.022)	0.706	0.077 (0.035, 0.118)	**<0.001**
By sex-specific quartiles [a]						
Q1	Ref.		Ref.		Ref.	
Q2	0.202 (0.008, 0.395)	**0.041**	0.083 (-0.011, 0.176)	0.083	0.122 (-0.021, 0.264)	0.095
Q3	0.287 (0.095, 0.479)	**0.003**	0.102 (0.007, 0.197)	**0.036**	0.185 (0.045, 0.326)	**0.010**
Q4	0.333 (0.142, 0.524)	**0.001**	0.063 (-0.030, 0.156)	0.184	0.269 (0.128, 0.409)	**<0.001**
**Subgroups by SUA levels**						
**Cut-points: clinical diagnosis criteria [b]**						
**Non-hyperuricemia group**						
SUA, +1 mg/dL	0.133 (0.060, 0.205)	**<0.001**	0.045 (0.008, 0.081)	**0.016**	0.102 (0.050, 0.154)	**<0.001**
Lower-level SUA (by sex-specific median)	Ref.		Ref.		Ref.	
Higher-level SUA	0.222 (0.083, 0.361)	**0.002**	0.086 (0.016, 0.155)	**0.016**	0.165 (0.064, 0.265)	**0.001**
**Hyperuricemia group**						
SUA, +1 mg/dL	-0.350 (-0.679, -0.021)	**0.037**	-0.237 (-0.395, -0.078)	**0.003**	-0.117 (-0.357, 0.123)	0.339
Lower-level SUA (by sex-specific median)	Ref.		Ref.		Ref.	
Higher-level SUA	-0.386 (-0.885, 0.113)	0.130	-0.294 (-0.555, -0.033)	**0.027**	-0.071 (-0.433, 0.291)	0.700
**Cut-points: sex-specific median**						
**Low-level SUA group**						
SUA, +1 mg/dL	0.149 (-0.020, 0.319)	0.084	0.038 (-0.048, 0.123)	0.388	0.134 (0.014, 0.255)	**0.029**
Lower-level SUA (by sex-specific	Ref.		Ref.		Ref.	

median)						
Higher-level SUA	0.203 (0.009, 0.397)	**0.040**	0.083 (-0.013, 0.178)	0.089	0.143 (0.003, 0.283)	**0.045**

(continued) Table S11. Associations between SUA and longitudinal cognition according to generalized estimating equations

Subgroups	Global cognitive function		Episodic memory		Executive function	
	**β (95% CI)**	***P***	**β (95% CI)**	***P***	**β (95% CI)**	***P***
**Cut-points: sex-specific median**						
**High-level SUA group**						
SUA, +1 mg/dL	-0.046 (-0.150, 0.057)	0.383	-0.073 (-0.125, -0.021)	**0.006**	0.030 (-0.045, 0.105)	0.434
Lower-level SUA (by sex-specific median)	Ref.		Ref.		Ref.	
Higher-level SUA	0.044 (-0.141, 0.229)	0.640	-0.037 (-0.133, 0.058)	0.441	0.083 (-0.050, 0.216)	0.223
**Cut-points: sex-specific 75th quantile**						
**Low-level SUA group**						
SUA, +1 mg/dL	0.138 (0.034, 0.243)	**0.010**	0.049 (-0.004, 0.102)	0.071	0.110 (0.036, 0.185)	**0.004**
Lower-level SUA (by sex-specific median)	Ref.		Ref.		Ref.	
Higher-level SUA	0.152 (-0.004, 0.309)	0.056	0.025 (-0.054, 0.103)	0.538	0.153 (0.040, 0.266)	**0.008**
**High-level SUA group**						
SUA, +1 mg/dL	-0.119 (-0.278, 0.039)	0.140	-0.110 (-0.187, -0.034)	**0.005**	-0.001 (-0.117, 0.115)	0.982
Lower-level SUA (by sex-specific median)	Ref.		Ref.		Ref.	
Higher-level SUA	-0.053 (-0.311, 0.205)	0.685	-0.058 (-0.189, 0.073)	0.386	0.013 (-0.174, 0.200)	0.892

Abbreviations: CI, confidence interval; β, Unstandardized beta coefficient; SUA, serum uric acid.; ref., reference.

Adjusted for: age, gender, marital status, education level, smoking status, drinking status, depressive symptoms, BMI, diabetes, dyslipidemia, stroke, heart-related diseases and prehypertension.

[a] The cutoff values were sex-specific quartiles of SUA (3.379, 3.979, and 4.716 mg/dL for women, 4.188, 4.964, and 5.841 mg/dL for men).

[b] The clinical diagnosis criteria for hyperuricemia were >6.0 mg/dL for women and >7.0 mg/dL for men

Table S12. Associations between changes in SUA and cognitive scores in 2018

	Global cognitive function		Episodic memory		Executive function	
	**β (95% CI)**	***P* value**	**β (95% CI)**	***P* value**	**β (95% CI)**	***P* value**
Non-hyperuricemia with elevated SUA	Ref.		Ref.		Ref.	
Non-hyperuricemia with decreased SUA	-0.414 (-0.722, -0.106)	**0.009**	-0.087 (-0.270, 0.096)	0.350	-0.369 (-0.583, -0.155)	**0.001**
Incident hyperuricemia	0.021 (-0.429, 0.471)	0.926	-0.090 (-0.358, 0.177)	0.508	0.032 (-0.281, 0.345)	0.841
Remittent hyperuricemia	0.016 (-0.885, 0.917)	0.972	-0.062 (-0.596, 0.472)	0.820	-0.035 (-0.661, 0.591)	0.912
Persistent hyperuricemia	-0.653 (-1.464, 0.157)	0.114	-0.139 (-0.620, 0.342)	0.570	-0.585 (-1.149, -0.022)	**0.042**
**Changes in SUA** [a]						
Changes in SUA, +1 mg/dL	0.079 (-0.038, 0.195)	0.187	0.010 (-0.059, 0.079)	0.773	0.064 (-0.017, 0.145)	0.121
By quartiles [b]						
Q1	Ref.		Ref.		Ref.	
Q2	0.101 (-0.248, 0.451)	0.569	0.052 (-0.155, 0.259)	0.624	0.129 (-0.114, 0.371)	0.298
Q3	0.500 (0.145, 0.856)	**0.006**	0.040 (-0.171, 0.251)	0.711	0.482 (0.235, 0.729)	**<0.001**
Q4	0.368 (0.008, 0.728)	**0.045**	0.045 (-0.168, 0.259)	0.676	0.307 (0.057, 0.557)	**0.016**

Abbreviations: CI, confidence interval; β, regression coefficient; SUA, serum uric acid.; ref., reference.

Adjusted for: age, gender, marital status, education level, smoking status, drinking status, depressive symptoms, BMI, diabetes, dyslipidemia, stroke, heart-related diseases, prehypertension, cognitive scores in 2015 and SUA levels in 2015.

[a] Changes in SUA was also calculated as the SUA level in 2015 minus that at baseline in 2011.

[b] The cutoff values were quartiles of changes in SUA (-0.073, 0.492, 1.150).

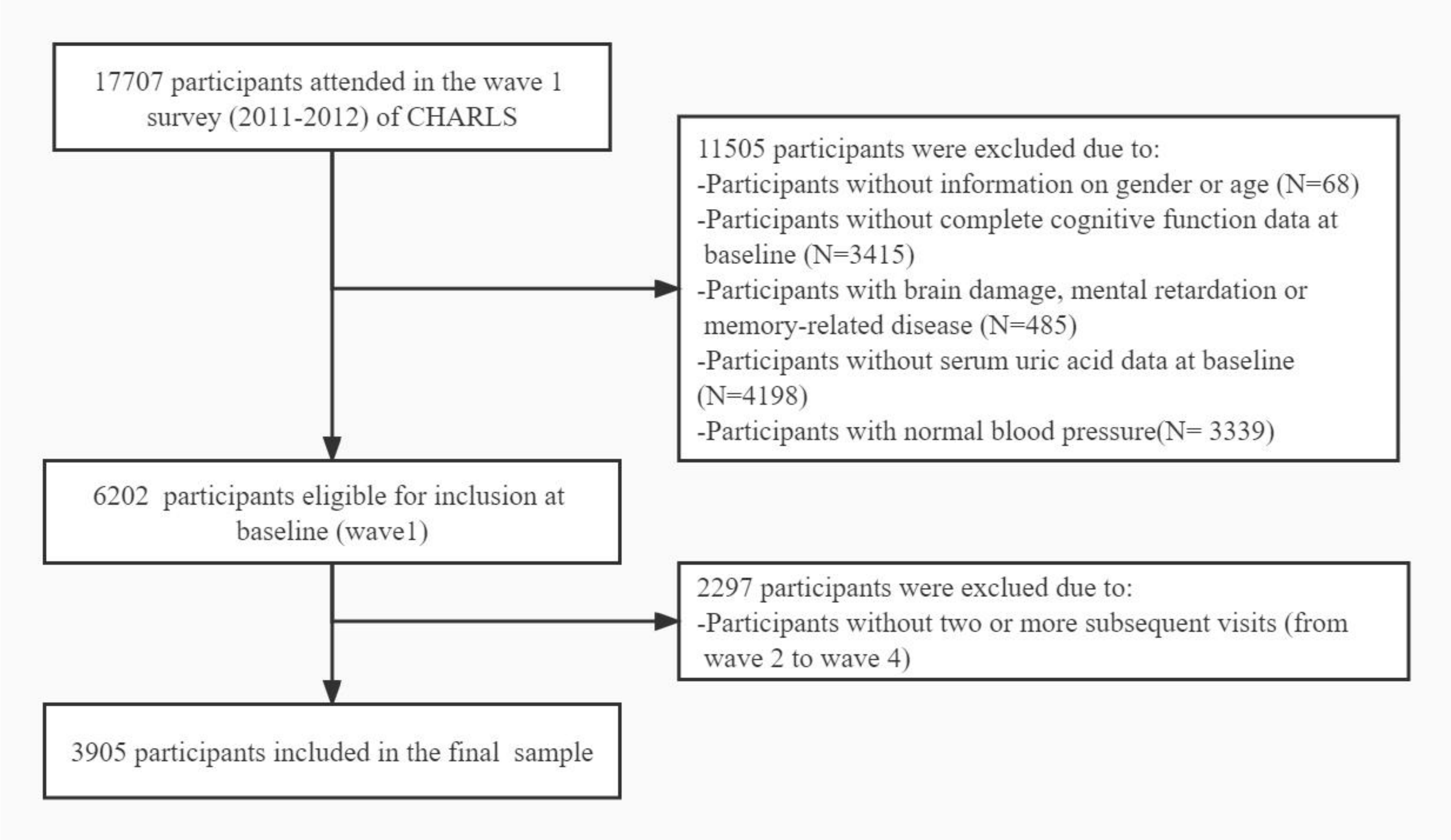

Figure S1. Study flowchart of participant selection in the first cohort

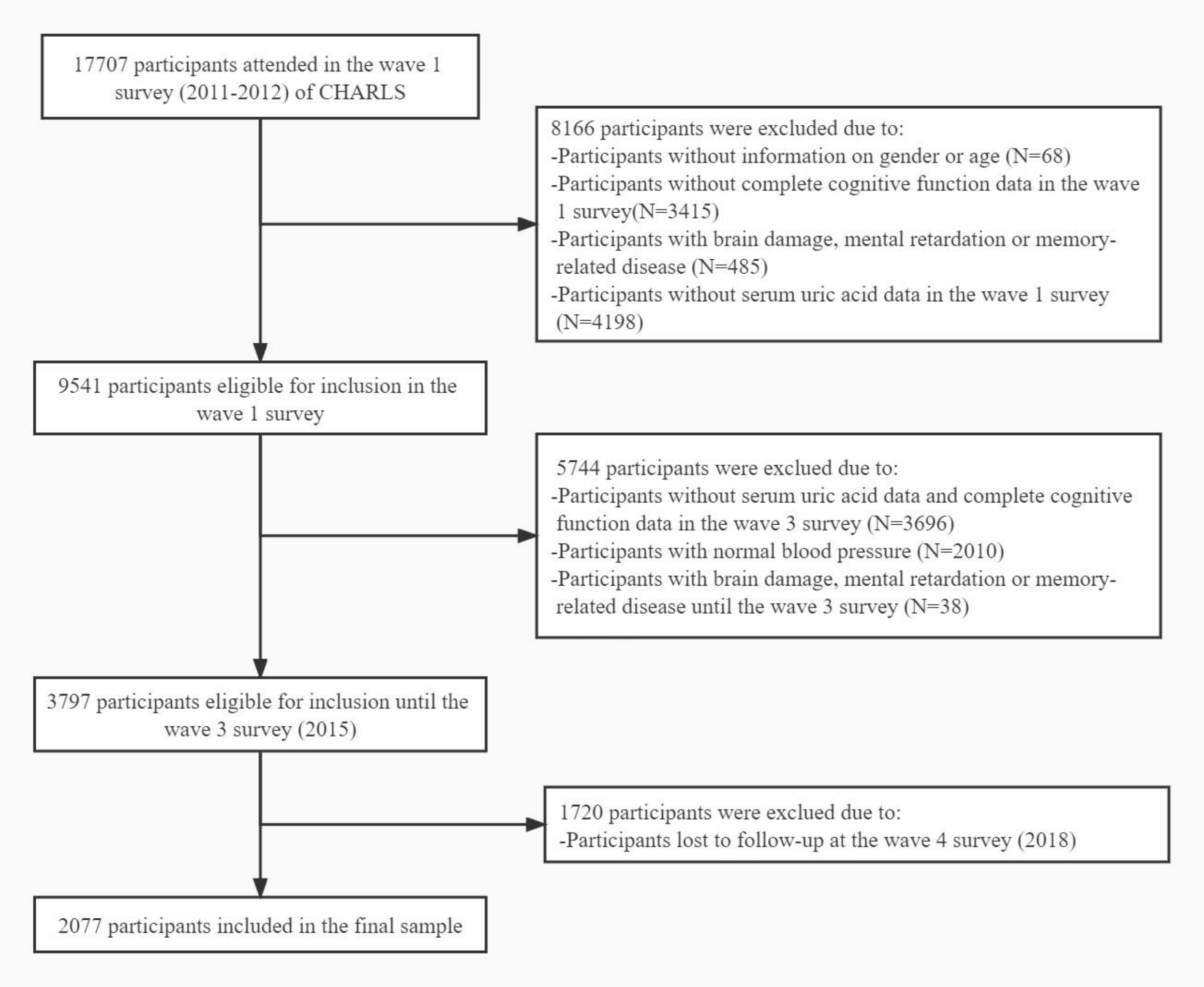

Figure S2. Study flowchart of participant selection in the second cohort

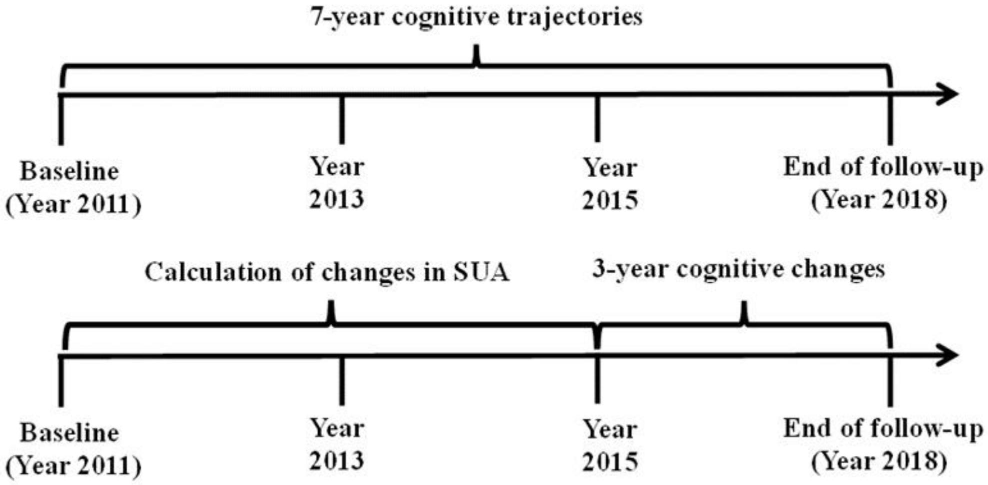

Figure S3. The time line of the study

Abbreviations: SUA, serum uric acid

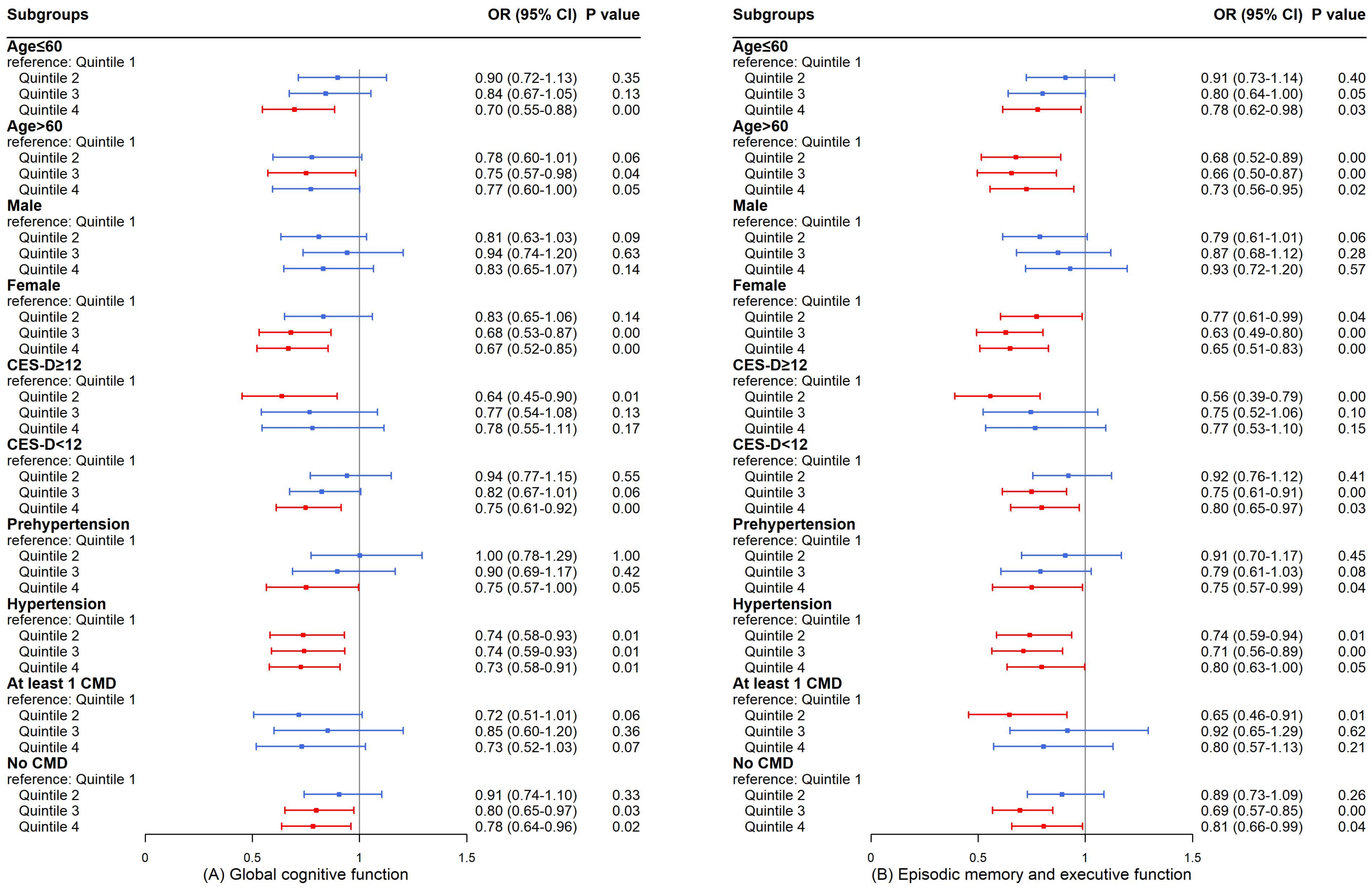

Figure S4. Associations between baseline SUA and cognitive trajectories in different subgroups

Graphs show OR and 95% CIs for the poorer cognitive trajectories. Red means OR value is statistically significant, and blue means OR value is not statistically significant.

Abbreviations: CI, confidence interval; OR, Odds ratios; SUA, serum uric acid; CMD, cardiometabolic diseases.

Adjusted for: age, gender, marital status, education level, smoking status, drinking status, depressive symptoms, BMI, diabetes, dyslipidemia, stroke, heart-related diseases and prehypertension.
